# Supplementary material for: Spatio-temporal heterogeneity of China’s import and export trade, factors influencing it, and its implications for developing countries’ trade
Source: PLoS One. 2024 Apr 18;19(4):e0300307. doi: 10.1371/journal.pone.0300307 (PMC11025959; doi:10.1371/journal.pone.0300307)
Supplement: S1 File — (PDF) [file pone.0300307.s005.pdf]

Dear editor:

We are delighted to have received your email. We have checked and revised the manuscript item by item according to the requirements as follows:

1. The list of authors and affiliations on the title page of my manuscript is correct, and our author contributions, competing interests and financial disclosures are correct.

2. We have removed the figures from the manuscript as requested and also standardised the naming of the figures and uploaded them separately as requested.

3. we have removed funding information from our manuscript, and ensured that the Financial Disclosure statement provided in the submission system is correct.

4. We have confirmed that our submission contains all the raw data necessary to replicate the results of our study.

5. We have updated the labelling of the chart files to ensure that each chart is easily identifiable by its chart number and can be easily associated with the corresponding chart title. The files are named "Fig 1", "Fig 2", etc.

6. We ensured all tables have citations.

7. We uploaded the LaTeX source file and the revised PDF.

8.We have downloaded the Author Formatting Checklist and checked

each item, and we have confirmed that the paper meets PLOS ONE's typographical requirements for references, tables and figures.

9. We have ensured that the figures uploaded have been evaluated by PACE and that our figures meet the technical requirements.
