# Supplementary material for: Spatio-temporal heterogeneity of China’s import and export trade, factors influencing it, and its implications for developing countries’ trade
Source: PLoS One. 2024 Apr 18;19(4):e0300307. doi: 10.1371/journal.pone.0300307 (PMC11025959; doi:10.1371/journal.pone.0300307)
Supplement: Attachment — Submitted filename: Reviewer Report.pdf [file pone.0300307.s006.pdf]

Review Report

The core claims are clearly expressed in abstract and conclusion part of the research paper. The empirical findings are cohere with each other and theorical framework of this work. The literature review is quite extensive and completed using up-to-date sources on the subject. The methods applied and the variables used in the methodology section of the article provided the required results. The findings of the article contributed to the literature as well. The findings and novelty of the manuscript contributes the existing literature. The English language of the article is clear and understandable, and the terminology used is compatible with the subject of the study. The article is very well-written and it is easy to follow. In addition, the article well discusses the topic of interest, and deals with a topic with many applications in practice. The quality of literature review and the aim of the paper is pretty well. The quality of the research is acceptable as well as the contribution of the paper. The quantitative research is very well conducted and the methodology described in the specific chapter is followed by current data processing. The methodology used is also very well described. The research fills a gap both by focusing on China's trade capacity and the influencing factors by considering the characteristics of spatial and temporal divergence to conduct exhaustive research on the influencing factors and temporal-spatial divergences in China's import and export trade. The researchers of the article recommend ensuring the optimization of trade channel layout. To enhance transportation efficiency, accessibility of goods, and decrease trade expenses, trade channel layout must be rationalized according to the spatial and temporal distribution traits of import and export trades. Firstly, upgrading of seaports and inland ports is essential for ameliorating goods' loading, unloading and transportation capacities, and accelerating trade transport times. On the other hand, diversifying and improving trade channels can be achieved through developing logistics networks, including highways and railroads. Additionally, facilitating faster and more convenient trade channels can be achieved by promoting the advancement of air freight and logistics technology. Strengthening trade cooperation and communication is also essential. This can be accomplished by actively expanding international trade partners and enhancing trade cooperation and communication with major trading partner countries and regions. Strengthening trade cooperation with developed economies is one approach to enhancing our trade level by learning advanced trade concepts and management experience. Additionally, our trade activities can be diversified and economic complementarity can be strengthened by focusing on trade cooperation with emerging market and developing countries. Furthermore, there is a need to establish and expand free trade zones and regional economic cooperation mechanisms to enhance trade facilitation and increase the scope of trade. Upgrading Trade

Technology: Enhancing trade-related technological research and innovation to analyze and forecast trade data more effectively. Utilize innovative technologies, such as big data analysis and artificial intelligence, to gain deeper insights into trade potential and predict trade demand and market fluctuations accurately. Additionally, strengthen trade information sharing and platform development to ensure timely and precise trade information. Furthermore, the emphasis should be on developing trade talent by enhancing training and exchange programs, as well as improving the technical capabilities and professional quality of practitioners. **In my opinion, the article has its merit and is of interest for the PLOS One readership. Consequently, the article is acceptable.**
