# Supplementary material for: Spatio-temporal heterogeneity of China’s import and export trade, factors influencing it, and its implications for developing countries’ trade
Source: PLoS One. 2024 Apr 18;19(4):e0300307. doi: 10.1371/journal.pone.0300307 (PMC11025959; doi:10.1371/journal.pone.0300307)
Supplement: Attachment — Submitted filename: Response to Reviewers.docx [file pone.0300307.s007.docx]

## Response to Reviewers

**Dear Editor:**

Thank you very much for your comments and professional advice.These opinions help to improve academic rigor of our article. Based on your suggestion and request, we have made corrected modifications on the revised manuscript. The figures in this article have been tested by PACE. Also, the “Supporting information” section is added as required, and the figures and tables are listed in the required format. Meanwhile, the manuscript had be reviewed and edited-by language services of ELSEVIER. We hope that our work can be improved again. In addition, **we adjusted the map, now using the natural earth map from the open source (http://www.naturalearthdata.com/).** Furthermore, we would like to show the details as follows:

1. In accordance with the journal's requirements, we downloaded all the templates, including the LaTeX template, and standardized our original article according to the template's requirements to ensure that our manuscript met PLOS ONE's stylistic requirements, including file naming requirements.

2. The data used in the manuscript are all from the relevant statistical departments in China, see the “Data sources” subsection of the manuscript, in order to ensure that the data are in line with the specification of the model calculation, we have rearranged the source data, and the organized data are sent in Excel files with attachments.

3. Corresponding author **Yuhuan Wu**：ORCID: https://orcid.org/**0009-0009-7218-8544**.

4. The first author **Haisong Wang**：ORCID: https://orcid.org/**0009-0009-6440-3961**.

5. The title on the online submission form and the title in the manuscript have been changed and are identical.

6. The author Ning Zhu has been added.

**7. The map is obtained from Natural Earth (http://www.naturalearthdata.com/). Natural Earth was built through a collaboration of many volunteers and is supported by NACIS (North American Cartographic Information Society), and is free for use in any type of project .**

8. **Copyright: ©** 2023 Wang et al. This is an openaccess article distributed under the terms of the Creative Commons Attribution License, which permits unrestricted use, distribution, and reproduction in any medium, provided the original author and source are credited.

9. **Data Availability Statement:** We have uploaded the minimal anonymized data.

10. **Funding:** Supported by Ministry of Education, Industry-University Cooperation

Collaborative Education Project (230825052507181). Funded by Science Research Project of Hebei Education Department (BJS2024097). Supported by Hebei Province Social Science

 Development Research Project (20230303051).

11. **Competing interests:** The authors have declared that no competing interests exist.

**The following is a response to the suggestions of all the esteemed reviewers**

**Reviewer #1**

Dear Authors,

Thank You for so relevant and exciting Article.

Please consider the Sustainability concept concerning the Article topic. I'd recommend considering ESG goal achievement in the Discussion section.

**The author’s response 1.1**

We have revised the Conclusions and Recommendations section to include the ESG objective under Implications for Developing Countries, and have adjusted the topic accordingly.

**Reviewer #2**

Your topic is very interesting Title: " Analysis of the Characteristics of Spatial and Temporal Divergence of China's Import and Export Trade ". I have some comments on your scripture which help you to update your manuscript.

Introduction

The introduction section doesn’t present clear picture what we need to need to learn, why we need to learn. Problem statement, research objectives or significance of the study should be clearly stated.

**The author’s response 2.1**

**We expand the Introduction to emphasize the importance of studying the spatial distribution of China's import and export trade and provide a reference for other developing countries; at the same time, the research content of this article is described in detail.**

Literature Review

The literature review section lacks theoretical underpinnings. In my opinion the whole section needs to overhaul. The researcher need to read past relevant studies and also make sure that you attempt to identify the gaps in the existing literature such that it becomes easy to justify and trace the contribution of your study. Moreover, scant studies helps you to identify the appropriate theory on which your relationship will be based.

**The author’s response 2.2**

**We rewrote the literature review as requested.**

Methodology

The study has critical deficiencies from a methodological standpoint. How the researcher determined the sample size? Where is the justification? In the current study there is no reference (Few but not related) to any sort of information about generating an item pool and receiving expert opinions for the scale. Moreover, what are the sources of scales? How it was developed? Unfortunately, it is a doubt whether the scales are valid. No information exits about a validity test for scales. Furthermore, How to overcome the common method bias as data has been collected from single method ? where is CFA ? THERE IS NO THEORETICAL GROUNDING.

**The author’s response 2.3**

**Based on the availability of data, the sample size of panel data and the selection of multiple linear regression data are determined, and some related references are added. The data come from the General Administration of Customs of China and the National Bureau of Statistics of China, so the data are real and reliable. Structural equation model is not used in this article. we have made some corrections, tests are carried out in exploratory spatial analysis and multiple linear regression, such as P-value test and R2 test.**

Discussion

The tests and presentation of findings are much more complicated. Again, I had difficulties in understanding the how findings of the study are connected to results of the analyses. Hypotheses testing is not well stated. The same applies to conclusion section.

**The author’s response 2.4**

**We have revised the conclusions based on the results of the model analysis and added conclusions for other developing countries.**

Contribution

The study's contribution is not written (a few of their but theoretical contribution is missing). Who will benefit from this research, both practically and theoretically? What role will it play in the existing literature? What's the latest/new?

**The author’s response 2.5**

**In the Introduction section, we add to the research contributions of this article.**

**Reviewer #3**

The core claims are clearly expressed in abstract and conclusion part of the research paper. The empirical findings are cohere with each other and theorical framework of this work. The literature review is quite extensive and completed using up-to-date sources on the subject. The methods applied and the variables used in the methodology section of the article provided the required results. The findings of the article contributed to the literature as well. The findings and novelty of the manuscript contributes the existing literature. The English language of the article is clear and understandable, and the terminology used is compatible with the subject of the study. The article is very well-written and it is easy to follow. In addition, the article well discusses the topic of interest, and deals with a topic with many applications in practice. The quality of literature review and the aim of the paper is pretty well. The quality of the research is acceptable as well as the contribution of the paper. The quantitative research is very well conducted and the methodology described in the specific chapter is followed by current data processing. The methodology used is also very well described. The research fills a gap both by focusing on China's trade capacity and the influencing factors by considering the characteristics of spatial and temporal divergence to conduct exhaustive research on the influencing factors and temporal-spatial divergences in China's import and export trade. The researchers of the article recommend ensuring the optimization of trade channel layout. To enhance transportation efficiency, accessibility of goods, and decrease trade expenses, trade channel layout must be rationalized according to the spatial and temporal distribution traits of import and export trades. Firstly, upgrading of seaports and inland ports is essential for ameliorating goods' loading, unloading and transportation capacities, and accelerating trade transport times. On the other hand, diversifying and improving trade channels can be achieved through developing logistics networks, including highways and railroads. Additionally, facilitating faster and more convenient trade channels can be achieved by promoting the advancement of air freight and logistics technology. Strengthening trade cooperation and communication is also essential. This can be accomplished by actively expanding international trade partners and enhancing trade cooperation and communication with major trading partner countries and regions. Strengthening trade cooperation with developed economies is one approach to enhancing our trade level by learning advanced trade concepts and management experience. Additionally, our trade activities can be diversified and economic complementarity can be strengthened by focusing on trade cooperation with emerging market and developing countries. Furthermore, there is a need to establish and expand free trade zones and regional economic cooperation mechanisms to enhance trade facilitation and increase the scope of trade. Upgrading Trade Technology: Enhancing trade-related technological research and innovation to analyze and forecast trade data more effectively. Utilize innovative technologies, such as big data analysis and artificial intelligence, to gain deeper insights into trade potential and predict trade demand and market fluctuations accurately. Additionally, strengthen trade information sharing and platform development to ensure timely and precise trade information. Furthermore, the emphasis should be on developing trade talent by enhancing training and exchange programs, as well as improving the technical capabilities and professional quality of practitioners. In my opinion, the article has its merit and is of interest for the PLOS One readership. Consequently, the article is acceptable.

**The author’s response 3.1**

**Thank you for your review and appreciation.**

**Reviewer #4**

Your work contains multiple analyses, is remarkable as such, and is gripping to read. My suggestions and editing requests for the study were sent to the editor as a PDF file. I wish you success in your future manuscripts.

**The author’s response 4.1**

**Thank you for your review and appreciation. We have revised the paper according to the PDF file you sent us.**

**Reviewer #5**

Although the subject of the article is attractive, there are many issues that need to be corrected in the article.

1-) The purpose of the article is not fully described in the introduction part of the article. What is the purpose of the article? Why is the topic of the article important? What is the article's contribution to the literature?

**The author’s response 5.1**

**We expand the introduction to emphasize the importance of studying the spatial distribution of China's import and export trade and provide a reference for other developing countries; at the same time, the research content of this article is described in detail.**

2-) Gini coefficient needs more detailed explanation.

**The author’s response 5.2**

**We provide a more detailed explanation of the Gini coefficient.**

3-) ESA and MLR also need more explanation.

**The author’s response 5.3**

**We provide a more detailed explanation of ESA and MLR.**

4-) Conclusion section is too short.

**The author’s response 5.4**

**We have expanded the Conclusion section of this article.**

5-) Is there no discussion section? The authors need to add a discussion section in the article.

**The author’s response 5.5**

**We add references to the import and export trade of other developing countries at last of this article.**

**We would like to thank the reviewers again for taking the time to review our manuscript.**

**Yours sincerely**

**Wang Haisong**

**Nov. 21, 2023**

**Department of Economics and Trade,**

**Hebei University of Water Resources and Electric Engineering,Cangzhou , Hebei**

**P.R.China**

**Tel: 86-0317-7587114**

**Email: micromoon@hbwe.edu.cn**
